# Cellulose Nanopaper: Fabrication, Functionalization, and Applications

**DOI:** 10.1007/s40820-022-00849-x

**Published:** 2022-04-13

**Authors:** Wei Liu, Kun Liu, Haishun Du, Ting Zheng, Ning Zhang, Ting Xu, Bo Pang, Xinyu Zhang, Chuanling Si, Kai Zhang

**Affiliations:** 1 Tianjin Key Laboratory of Pulp and Paper, Tianjin University of Science and Technology, Tianjin, 300457 People’s Republic of China; 2 Sustainable Materials and Chemistry, Department of Wood Technology and Wood-Based Composites, University of Göttingen, 37077 Göttingen, Germany; 3 Department of Chemical Engineering, Auburn University, Auburn, AL 36849 USA; 4 Department of Automotive Engineering, Clemson University, Greenville, SC 29607 USA

**Keywords:** Nanocellulose, Cellulose nanopaper, Cellulose nanocrystals, Cellulose nanofibrils, Cellulose nanomaterials

## Abstract

Cellulose nanopaper has shown great potential in diverse fields including optoelectronic devices, food packaging, biomedical application, and so forth, owing to their various advantages such as good flexibility, tunable light transmittance, high thermal stability, low thermal expansion coefficient, and superior mechanical properties. Herein, recent progress on the fabrication and applications of cellulose nanopaper is summarized and discussed based on the analyses of the latest studies. We begin with a brief introduction of the three types of nanocellulose: cellulose nanocrystals, cellulose nanofibrils and bacterial cellulose, recapitulating their differences in preparation and properties. Then, the main preparation methods of cellulose nanopaper including filtration method and casting method as well as the newly developed technology are systematically elaborated and compared. Furthermore, the advanced applications of cellulose nanopaper including energy storage, electronic devices, water treatment, and high-performance packaging materials were highlighted. Finally, the prospects and ongoing challenges of cellulose nanopaper were summarized.

## Highlights

- Preparation strategies of cellulose nanopaper were elaborated.
- Functionalization of cellulose nanopaper and its advanced applications were summarized.
- Prospects and challenges of cellulose nanopaper were discussed.

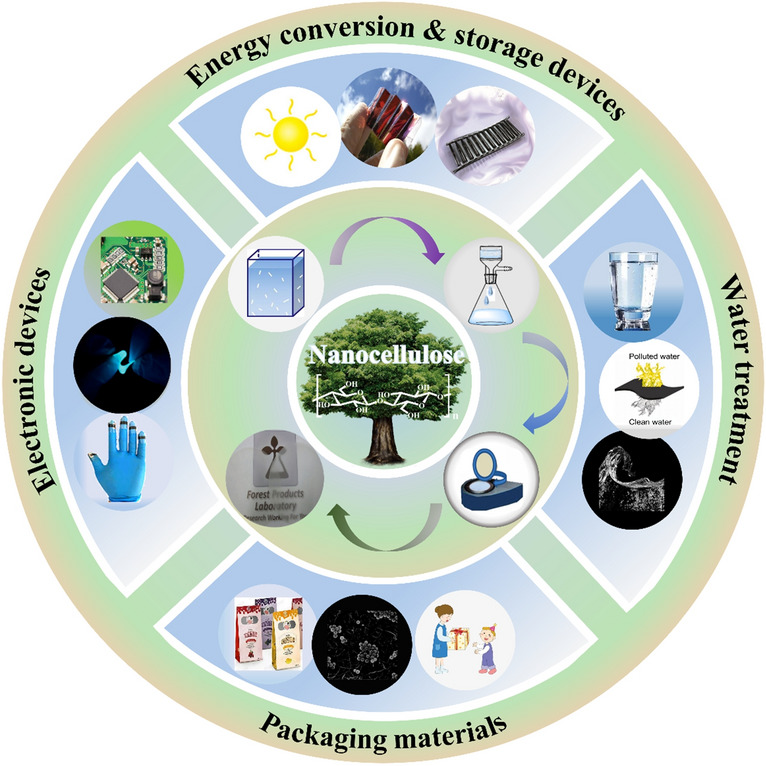

## Introduction

**Fig. 1:**
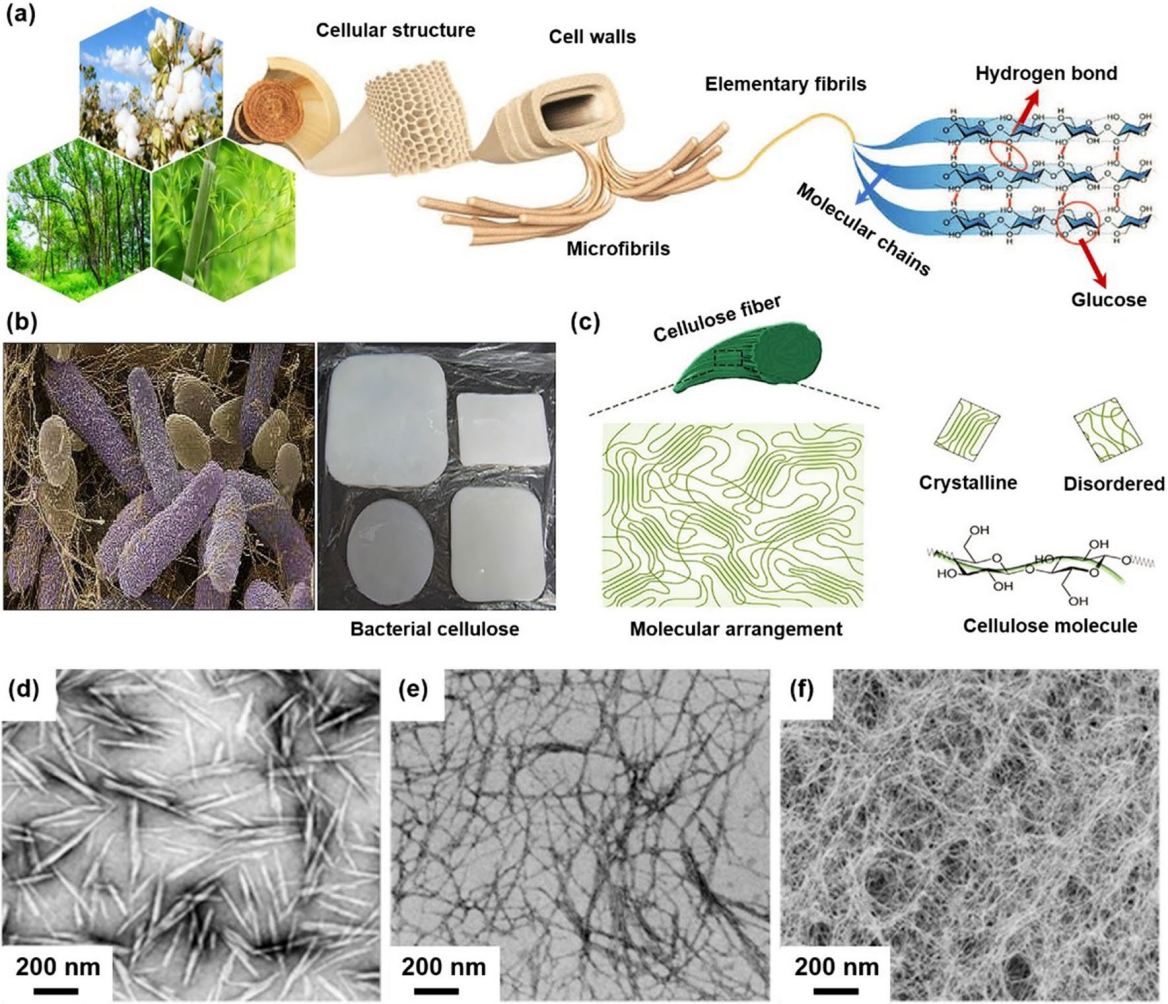
**a** Schematic illustration of hierarchical fibril structure and morphologies of lignocellulose [45]. **b** SEM image of Acetobacter xylinus, optical photograph of BC and formation of BC [46]. **c** Schematics of the crystalline and disordered regions [45]. **d** TEM image of CNCs [47]. **e** TEM image of CNFs [48]. **f** SEM image of BC [4]

As the most abundant natural polymer, cellulose is the main structural component of plant’s cell wall (Fig. 1a, c) and many microorganisms (such as fungi, bacteria, and algae) [1, 2]. Cellulose is composed of β-D-glucopyranose units linked by β-(1–4) glycosidic bonds [3–5]. Cellulose with a one-dimensional scale between 1 and 100 nm is called nanocellulose, which has the chemical structure characteristics of original cellulose and has received extensive scientific and technological attention in the past two decades [6, 7]. Nanocellulose not only possesses intrinsic nature of cellulose such as biodegradability, renewability, and ease of chemical modification, but also has outstanding characteristics, such as nanoscale effects, high tensile strength and elastic modulus (130–150 GPa), high specific surface area (up to several hundreds of m^2^·g^−1^), and low density (1.6 g cm^−3^) [8]. Owing to such excellent properties, nanocellulose has shown great potential in many emerging applications, such as biomedical materials [9–13], paint and coatings [14, 15], sensors [16–18], photonic and electronic devices [19–23], and energy storage materials [24–28].

The pulping and bleaching technology of modern pulp and paper mills could be amenably upgraded for nanocellulose production, which also provides a new development strategy for traditional pulp and paper enterprises and open up a valuable path for the high-value utilization of various biomass resources [29–31]. Cellulose nanopaper (CNP), a kind of film material mainly constructed from nanocellulose, has superior physical–chemical properties, such as tunable optical transmittance, high thermal stability, low thermal expansion coefficient (< 8.5 × 10^–6^ K^−1^), and good mechanical properties (high modulus of 7.1–14 GPa) [32–34]. Moreover, combining nanocellulose with other functional nanomaterials would endow the CNP with diverse new functionalities, leading to the expansion of the applications from traditional areas to high-tech fields such as electronic devices, clean energy, biomedicine, and water treatment [35–39].

Several reviews have summarized the development of CNP for various applications, to which the readers are directed for additional information [40–44]. However, a comprehensive review about the fabrication, functionalization and emerging applications of CNP is still missing. In this review, based on the analysis of the latest research, we summarize and discuss the latest developments in the preparation, functionalization, and application of CNP. Firstly, the differences in preparation and properties of cellulose nanocrystals, cellulose nanofibrils, and bacterial cellulose are briefly introduced. Then the manufacture of CNP and its various applications, especially applications in emerging fields such as energy storage, electronic equipment, water treatment, and high-performance packaging materials, are deeply discussed. Finally, the prospects and challenges of CNP are summarized.

## Fabrication of Cellulose Nanopaper

### Classification, Preparation, and Performance of Nanocellulose

According to the size, morphology, preparation techniques, and sources, nanocellulose can be classified into three main categories: (1) Cellulose nanocrystals (CNCs), also known as nanocrystalline cellulose (NCC) or cellulose nanowhiskers (CNWs), are rigid rod-like particles of 10–30 nm in diameter and less than 500 nm in length (Fig. 1d) [49–51]. CNC is generally obtained by hydrolysis of cellulose raw materials with strong inorganic acid or enzyme [52, 53]. In the process of hydrolysis, the amorphous area of cellulose is degraded into sugar, and the crystalline area is retained, and finally whisker-like or spherical CNC is obtained [53, 54]. Compared with cellulose raw materials, CNCs have a higher degree of crystallinity ranging from 54 to 88% [55]. (2) Cellulose nanofibrils (CNFs), also termed as nanofibrillated cellulose (NFC) or microfibrillated cellulose (MFC), consist of both individual and aggregated nanofibrils (Fig. 1e) [56, 57]. The elementary fibril has a diameter of 3–5 nm and a length of 500–1000 nm, while the aggregates are in the range of 20–50 nm in diameter [58]. CNF is mainly obtained by treating cellulose raw materials with strong mechanical shear force [59]. During the treatment, the amorphous region of cellulose is usually not removed. CNF is composed of both crystalline region and amorphous region, with large aspect ratio and good flexibility [60, 61]. (3) Bacterial cellulose (BC), a kind of highly crystalline cellulose without lignin and hemicellulose, is mainly produced by Acetobacter species via a biotechnological assemble processes using low-molecular weight carbon sources, e.g., D-glucose, as feedstock (Fig. 1b, f) [62, 63]. Depending on the type of bacteria and the culturing conditions, the typical BC fibril has a diameter of 20–100 nm and a length of several microns and could be processed into various shapes such as spheres and membranes per different fermentation conditions [64]. BC is usually synthesized by a specific bacterium (e.g., glucoacetobacter). Glucose chains are generated in the bacterial cell body and extruded from the pores of the cell membrane. Several glucose chains further form microfibrils, which are intertwined to form a three-dimensional nanonetwork, namely BC.

As discussed above, different types of nanocellulose exhibit different properties, which further determines different end applications. For instance, CNCs with high crystallinity, high thermal stability, and unique chiral nematic structure could be more suitable to be used as reinforcing nanofillers for nanocomposites and to make iridescent films [65]. However, when compared to CNF and BC, the self-assembled nanostructure (e.g., film) of CNC is very brittle due to the lack of an energy-dissipating amorphous phase and its inability to form entangled networks [66]. Thus, it would be more reasonable to select CNF and BC for the fabrication of mechanically robust nanostructures such as nanopaper, foams, and aerogels.

### Preparation Strategies of Cellulose Nanopaper

In 1992, Revol and coworkers firstly fabricated CNP by simply evaporating water from the aqueous CNC suspensions [67]. This work mainly focused on the chiral nematic liquid crystalline phase and the twisted fibrillary layers solidified from CNCs. Later, Dufresne et al. reported the fabrication of CNP by casting method with sugar beet cellulose nanofibers [68]. Taniguchi and coworkers prepared CNP with nanofibers from different types of natural fibers such as wood pulp fibers, tunicin cellulose, and silk fibers [69]. The obtained films with a thickness of 3–100 μm were strong, homogeneous, and translucent. In these studies, CNP exhibited much higher tensile strength than commercial print grade papers. The physiochemical properties of the resultant CNP are affected by preparation methods, filter media, drying methods, and so forth [70–72]. However, the effects of such parameters on the self-assembly of nanocellulose during the preparation procedure have not been fully understood. Isogai et al. compared the CNP manufactured by CNFs with larger and smaller (< 10 nm) fibril dimeter and found that CNP prepared from finer CNFs has a higher transparency (90%), and higher tensile strength and Young's modulus (233 MPa and 6.9 GPa, respectively) [73]. Aulin et al. used dispersion casting method and coating method on base paper to prepare carboxymethyl microfibrillated cellulose (MFC) film, and the effects of relative humidity on the oxygen barrier properties of nanopaper were studied [74]. The results showed that the CNP had better oxygen barrier performance at lower humidity. Currently, there are three main methods including suction filtration, casting, and coating to produce CNP.

#### Filtration Method

**Fig. 2:**
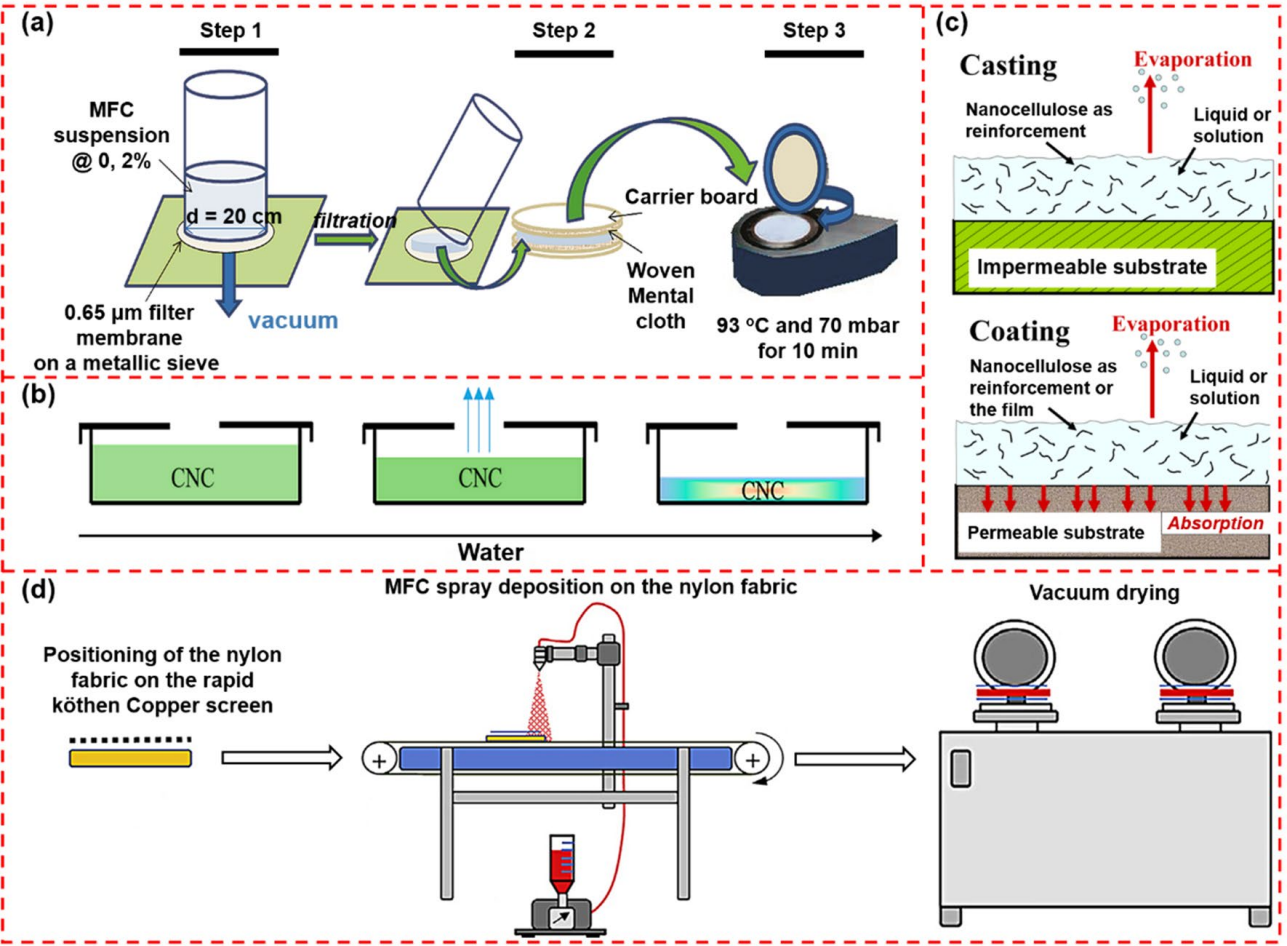
Schematic illustration of the preparation procedure of CNP via **a** filtration method [75] and **b** casting method [76]. **c** Schematic illustration of the difference in how the liquid is withdrawn during the casting and filtration method [77]. **d** Schematic illustration of the fabrication of CNP by spraying deposition [78]

The suction filtration method has been frequently used for preparing CNP in laboratory-scale setups (Fig. 2a). The main steps involved can be briefly described as: the nanocellulose suspension is firstly diluted to a concentration of 0.01–1.0 wt.%. After thorough mixing, the nanocellulose suspension is poured into a vacuum or pressure filtration device [79, 80]. As the filtration progresses, most of the free water is removed and nanocellulose suspension forms a dense packing layer in the bottom filter. The increase in the nanocellulose concentration induces the aggregation of nanocellulose to form a wet nanopaper. After that, the wet nanopaper is peeled from the filter and CNFs will be finally obtained after pressing and drying [81]. Sehaqui et al. developed a rapid procedure to fabricate nanopaper, involving vacuum filtration, wet paper web transfer, and vacuum drying [79]. The whole process could be completed within 1 h, indicating the great enhancement of the preparation efficiency. As verified by many studies, the size of the filter used to prepare CNP is a crucial parameter to be considered. The pore size of the traditional wire sieve is much larger than the size of nanocellulose, resulting in a low retention rate of nanocellulose. Although the use of filters with a pore size in the range of 0.1–0.65 μm or smaller can greatly reduce the loss of nanocellulose, the water removal procedure could last for several hours or even several days, which greatly reduces the viability for the large-scale production. It was shown that polyelectrolytes could be used as filter aids to improve the dewatering performance of nanocellulose suspensions. Wetterling et al. developed an electro-assisted filtration method to promote the dewatering of cellulosic materials [82]. The results demonstrated that the ionic strength had a crucial effect on the electro-assisted filtration process. Electro-assisted filtration was found to improve the dewatering rate of the studied cellulosic material, and the potential improvement compared to pressure filtration increased with the specific surface area of the solid material. Higher power is required for the electro-assisted filtration of the system with higher ionic strength.

#### Casting Method

Compared with the suction filtration, the casting method can not only achieve a 100% retention of nanocellulose, but also result in CNP with specific microstructures by fine-tune the evaporating condition (Fig. 2b) [74]. In this method, the evaporation of the solvent usually takes several hours, during which nanofibers gradually get closer to each other, thereby increasing the interaction between the nanofibers and finally agglomerating to obtain the nanopaper. Based on this method, researchers developed a semi-industrial roll-to-roll pilot line, realizing the continuous production of CNP [83]. It is worth noting that such roll-to-roll strategy can only process CNFs-based CNP. CNCs are of lower aspect ratio and higher rigidity, thus not easy to form a continuous film through the roll-to-roll pilot line without aiding agents.

#### Coating Method

Unlike the casting method which processes CNP on an impermeable substrate, coating approach employs a permeable substrate, permitting the nanocellulose to deposit on it (Fig. 2c). Generally, the coating method is mainly to coat nanocellulose on a substrate with a porous surface and then remove the excess paint through a blade. After drying, a composite nanopaper consisting of a nanocellulose-formed top layer and an underlying substrate can be obtained [77]. Such nanopapers have excellent light transmittance and low oxygen permeability, demonstrating great potential as environmentally friendly barriers [84]. Compared with pure CNP, nanocomposite coatings are highly versatile, enabling CNP to combine excellent optical transmittance, high hardness, fracture toughness, and hydrophobic surface properties [85].

However, to achieve large-scale production, the following challenges need to be overcome in the coating method: (1) Nanocellulose has inherent characteristics of small size, large specific surface area, high hydrophilicity, and good water retention, leading to issues of low retention rate and dehydration difficulty during the CNP forming process. To tackle these issues, several approaches such as adding retention aids, plasticizers, or compatibilizers with nanocellulose have been developed to improve the retention rate and coating properties of nanocellulose suspensions [85]. (2) Due to the high water retention rate of nanocellulose, the efficiency of the traditional coating process is relatively low. To accelerate the dewatering process, Beneventi et al. reported a more efficient way by spray deposition of high concentrated CNFs suspension on a wet porous nylon fabric to fabricate nanopaper with excellent barrier properties and high mechanical properties (Fig. 2d) [78]. Similarly, Krol et al. used the spray deposition to fabricate CNFs-SiO_2_ composite nanopapers successfully [86]. For silica mass fraction above 20%, SiO_2_ clusters induced a net increase in air permeability and ionic conductivity up to 12 nm^2^ and 1.5 mS cm^−1^ for a SiO_2_ content of 33%. Shanmugam et al. reported that in terms of tensile index, the nanopaper prepared by the spraying method showed comparable performance to the nanopaper fabricated by the filtration method [87].

#### Emerging Methods

To accomplish high performance in nanopaper, many novel strategies have been proposed. For instance, Kumar et al. developed a roll-to-roll coating process to deposit CNFs suspension on packaging cardboard via a slot die followed by infrared and air drying. This method provided a continuous and industrially compatible process to reinforce the commercial paperboard with CNF coatings with high strength and barrier properties [88]. Chowdhury et al. further investigated the roll-to-roll coating process and found that, by simply adjusting the concentration of CNCs suspension, the anisotropy of CNCs can be achieved and controlled, which in turn affected the coating properties, e.g., the water vapor transmission rate in particular [89]. Azrak and co-workers developed an efficient approach for continuous processing of CNP through traditional single-screw extrusion [90]. It was demonstrated that highly loaded CNF/processing aid pastes (91 wt% CNF, ≤ 9 wt.% of processing aid e.g., carboxymethyl cellulose) with a solid content of 25% could be extruded at rates of up to 7.45 ± 0.47 kg/h, which significantly reduced the preparation and drying time. Intriguingly, the extruded CNP exhibited equivalent mechanical properties when compared to the CNP prepared by the conventional solution casting approach.

## Functionalization of Cellulose Nanopaper

Due to the high hydrophilicity, CNP is mechanically strong in dry conditions, but weakened in a high humidity environment [91, 92]. This downside limits CNP’s practical application under all-around scenarios. The water resistance of CNP can be improved by compounding nanocellulose with hydrophobic materials prior to the formation of CNP or directly hydrophobilizing the pre-formed CNP surface [93]. For example, Cunha et al. demonstrated a facile approach to prepare moisture-stable CNP by topochemical acetylation [94]. It was found that the acetylated CNP sorbs as little as 2.0–2.3% moisture at 53%RH, which is much lower than the untreated sample (3.8–4.8%). Operamolla and co-workers reported the fabrication of hydrophobized CNP by dipping it in a dichloromethane solution of lauroyl chloride and pyridine [95]. Results indicate that the hydrophobized CNP has a more compact surface morphology than the starting CNP and exhibits significantly enhanced resistance to water. Zhu et al. treated the CNP with glutaraldehyde and obtained the modified CNP with excellent dimensional stability in water [96]. This shape-stable and transparent CNP was further used as the substrate to fabricate gravure printed antenna. In another example, Jung and co-workers reported a simple and effective way to enhance the water resistance by treating the CNP with 0.1 M octadecyltrichlorosilane for 30 s [97]. It was discovered that the contact angle of the CNP was significantly increased from 58° to 103° after treatment. The authors further demonstrated that the modified CNP could be used as promising substrate for solar cells.

In addition, enhanced performance and/or novel functionalities can be introduced into CNPs, extending their applications in value-added fields [98, 99]. In the following subsections, we will introduce a variety of emerging composite nanopapers with functional nanomaterials (e.g., nanomaterials with 0D, 1D, or 2D structure, macromolecular polymers, etc.) in recently.

### Nanomaterials Functionalized Nanopaper

**Fig. 3:**
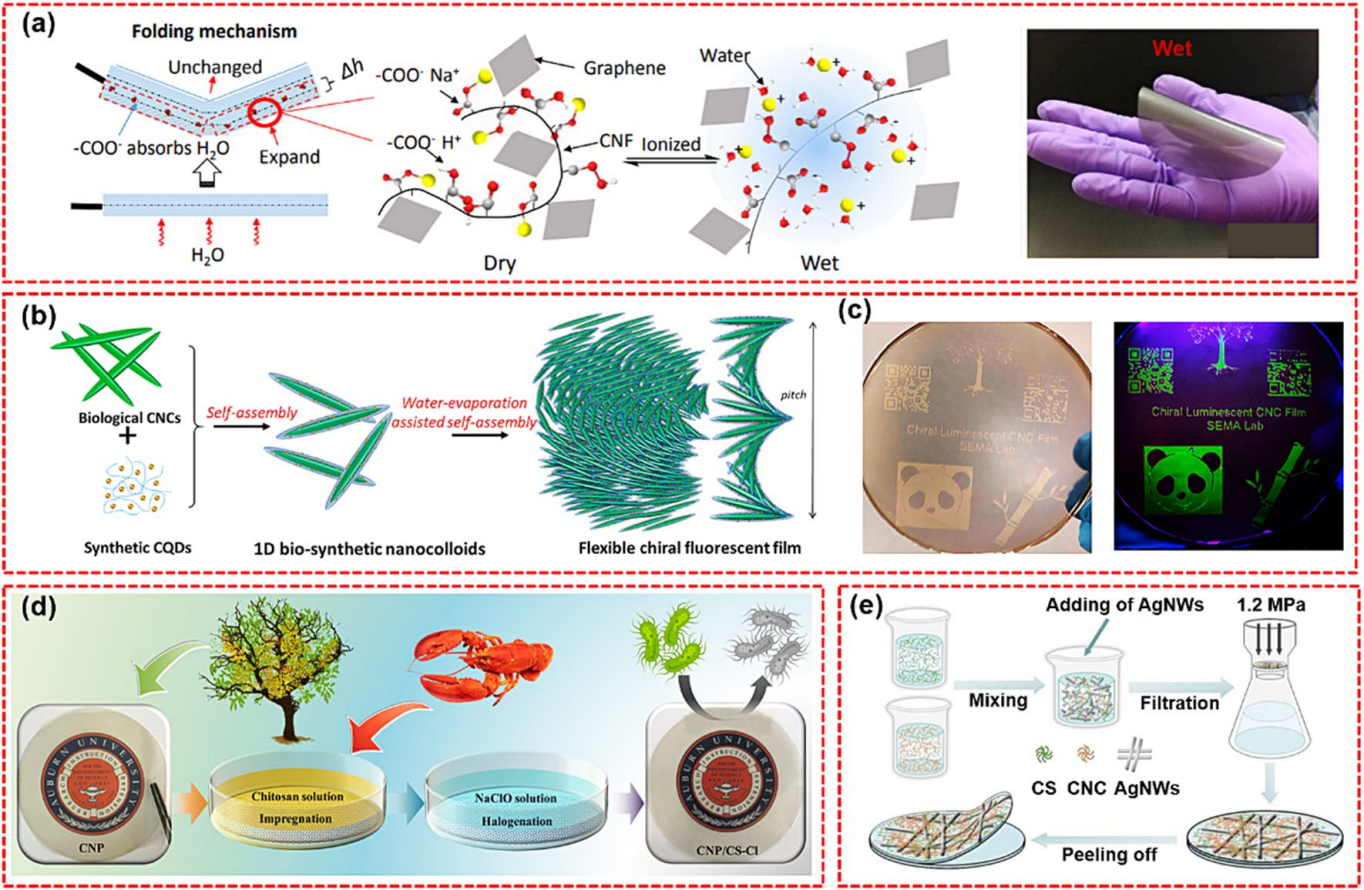
**a** proposed folding mechanism and bending photograph of graphene/CNFs nanopaper [100]. **b** Self-Assembly of Chiral Fluorescent CQD/CNCs Nanostructures. **c** Patterned chiral luminescent CNCs films with 10 cm diameter under natural light (left) and at 365 nm UV light (right) [101]. **d** Schematic illustration of the functionalization of CNP by impregnation of CS and the followed halogenation [102]. **e** Schematic illustration of the assembling procedures for CNCs/CS/AgNWs TCCNP [103]

The integration of nanocellulose with various metal nanoparticles endows the resultant CNP with new functionalities and extends their application to unprecedented fields, e.g., sensors and catalysis [104]. For example, nanopaper possessing magnetism, nanosurface roughness, and excellent mechanical properties was obtained by incorporating Fe_3_O_4_ nanoparticles (0D) to nanocellulose CNP. Such magnetic nanopaper hold the promise to prepare magnetoelectronic devices [105]. Owing to the excellent flexibility of CNP and high conductivity of silver nanowires (AgNWs), the two constituents have been combined in transparent and conductive nanopaper [106]. The assembly of nanocellulose and other inorganic nanomaterials has become a new strategy for the development of high-performance nanopapers. For example, antibacterial property was accomplished by integrating ZnO in CNP, CNP possessing adjustable thermal conductivity was obtained by compositing sepiolite nanofibers (a kind of nanosilicate with fiber morphology of about 1–5 μm in length and 50–100 nm in diameter) with nanocellulose [107]. Layered materials (2D) such as graphite, layered metal oxides and layered silicates can be exfoliated into few- or even single-layered sheets. CNP made of nanocellulose with these two-dimensional nanomaterials has demonstrated great potential in various emerging fields such as electronics and energy devices. For instance, Xu et al. used a simple high-speed blending technology, in the presence of amphiphilic CNFs, and exfoliating graphite at the same time, the graphene can be dispersed in water in one step, and the prepared aqueous suspension is made into a flexible and lightweight graphene/CNFs paper by vacuum filtration [100]. As shown in Fig. 3a, this nanopaper showed rapid deformation or bending motion in the presence of moisture, and instantaneous recovery in the absence of moisture stimulation, simulating the closing or opening behavior of shame plants or the movement of soft robots. MXenes, two-dimensional transition metal carbides, exhibit excellent properties in many fields such as energy storage and sensor. Song et al. used a simple vacuum-assisted filtration method to prepare a flexible cellulose nanofiber (CNFs)/Ti_3_C_2_ composite membrane [108]. The synthesized Ti_3_C_2_/CNFs composite films presented outstanding flexibility because the films can be fold to any shape, which enables Ti_3_C_2_/CNFs composite films to be the potential application in flexible electronic devices for heat dissipation.

Berglund et al. fabricated fire-resistant CNP by mixing nanoclay with CNFs suspension [109]. Hu and coworkers successfully fabricated CNP with superior thermal and mechanical properties by combing CNFs with layered boron nitride nanosheets [110]. The same group also designed a double-layer nanopaper with excellent thermal conductivity, electrical insulation, and transparency by coating a thin layer of boron nitride on the surface of nanopaper [111]. As shown in Fig. 3b, Xiong et al. reported the emission of needle-like nanostructures by self-assembly of CNCs decorated with carbon quantum dots (CQDs) [101]. The chiral CQD/CNCs film exhibits a strong iridescent appearance and enhanced luminescence superposition, which is significantly higher than that of the CQD film and other reported CQD/CNCs films (Fig. 3c).

### Polymer Functionalized Nanopaper

The combination of polymers and nanocellulose provides a solution to enhance the performances of nanopapers including improved mechanical and optical properties, lower surface roughness, and higher water resistance [112]. Sehaqui et al. reported the fabrication of CNP by combing 2-hydroxyethyl cellulose (HEC) with CNFs suspension. Compared with CNFs-based CNP, the HEC-CNFs CNP possessed higher elongation at break and lower tensile strength [79]. Wang et al. proposed a simple method for bulk gradient nanocomposites of CNFs and custom-synthesized copolymers [113]. CNFs/copolymer mixtures with different weight ratios were directly written into filaments adjacent to each other in a stripe pattern, and then, the filaments were continuously dried to prepare CNP with mechanical gradient properties. This functionalized CNP showed great promise for stretchable electronic devices. Most recently, chitosan (CS) has been proven as a promising material for the functionalization of CNP to achieve multifunctional properties such as antibacterial ability, water resistance, and improved mechanical strength and barrier performance [114, 115]. As shown in Fig. 3d, Du et al. functionalized CNP by impregnation of CS and the followed halogenation [102]. It was found that the functionalized CNP exhibited improved mechanical strength at both dry (38.3%) and wet (512.6%) state, enhanced barrier properties towards both oxygen and water vapor, as well as excellent antibacterial performance against both *S. aureus* and *E. coli*. As illustrated in Fig. 3e, Zhang et al. used CS and TEMPO-oxidized CNCs as the fiber backbone, and AgNWs as the conductive network and successfully designed flexible transparent conductive TCCNP [103]. The results showed that with the addition of CS, the mechanical stress of TCCNP increased from 14.5 to 30.8 MPa, and it had good transparent conductivity, and the sheet resistance is 4.43 Ohm sq^−1^. This discovery will open the way for the further development of high-end flexible paper electronic technology using high-performance polysaccharide-based TCCNP.

## Applications of Cellulose Nanopaper

Owing to the excellent properties, such as superior mechanical properties (high modulus, high tensile strength), high thermal stability, low thermal expansion coefficient, and easy adjustment of optical properties, CNP has been widely used in many emerging fields including electronic devices, energy storage, packaging materials, water treatment and so forth.

### Electronic Devices

Recently, various electronic devices are highly demanded due to the rapid development of the electronic information society. Owing to the inherent advantages such as lightweight, wide range of sources, and excellent mechanical properties, CNP has shown great potential in the design and manufacturing of electronic devices [116–118].

#### Conductive Film Materials

Many types of conductive nanopaper based on nanocellulose have been developed. Su et al. prepared a transparent conductive nanopaper by assembling polydopamine-modified nanocellulose with AgNWs [119]. The resultant nanopaper displayed the high transmission (90.93% at the wavenumber of 550 nm), good mechanical properties, and excellent corrosion resistance. Also, Hoeng et al. developed a flexible conductive film by coating CNFs/AgNWs suspension on polyetherimide (PEI) film [120]. The results showed that performances combining a sheet resistance of 13 ± 3 Ω sq^−1^ with a transparency of 90.1 ± 0.5% at 550 nm were achieved without any additional post-treatment to the film. Additionally, nanocellulose can also be processed into highly conductive composite by combining CNP with highly conductive materials, such as carbon nanotubes and graphene [121, 122]. Compared with the commercialized ITO/polyethylene terephthalate (PET) transparent conductive paper, such CNP had high thermal stability, renewability, biodegradability, and low thermal expansion coefficient, showing great potential in the new generation of electronic devices. Cao et al. reported the preparation of highly flexible CNF/MXene composite nanopaper through a vacuum-filtration-induced self-assembly process. The obtained CNF/MXene nanopaper exhibited a nacre-like lamellar structure, resulting in high tensile strength (135.4 MPa) and excellent folding endurance (14,260 times). Moreover, the composite nanopaper showed high electrical conductivity (739.4 S m^−1^) and excellent specific EMI shielding efficiency (2647 dB cm^2^ g^−1^). Most recently, Liu et al. reported the fabrication of CNFs/AgNWs nanopaper through a step-by-step self-assembly process [123]. It was found that the obtained CNFs/AgNWs nanopaper showed a unique layered structure and improved two-sidedness. The optimized sample exhibited an excellent mechanical strength (98.6 MPa) and a high conductivity (1673 S cm^−1^) simultaneously. As a result, the CNFs/AgNWs nanopaper was proved as a promising EMI shielding material with a high shielding effectiveness of up to 67.27 dB in the X band.

#### Electronic Skin

**Fig. 4:**
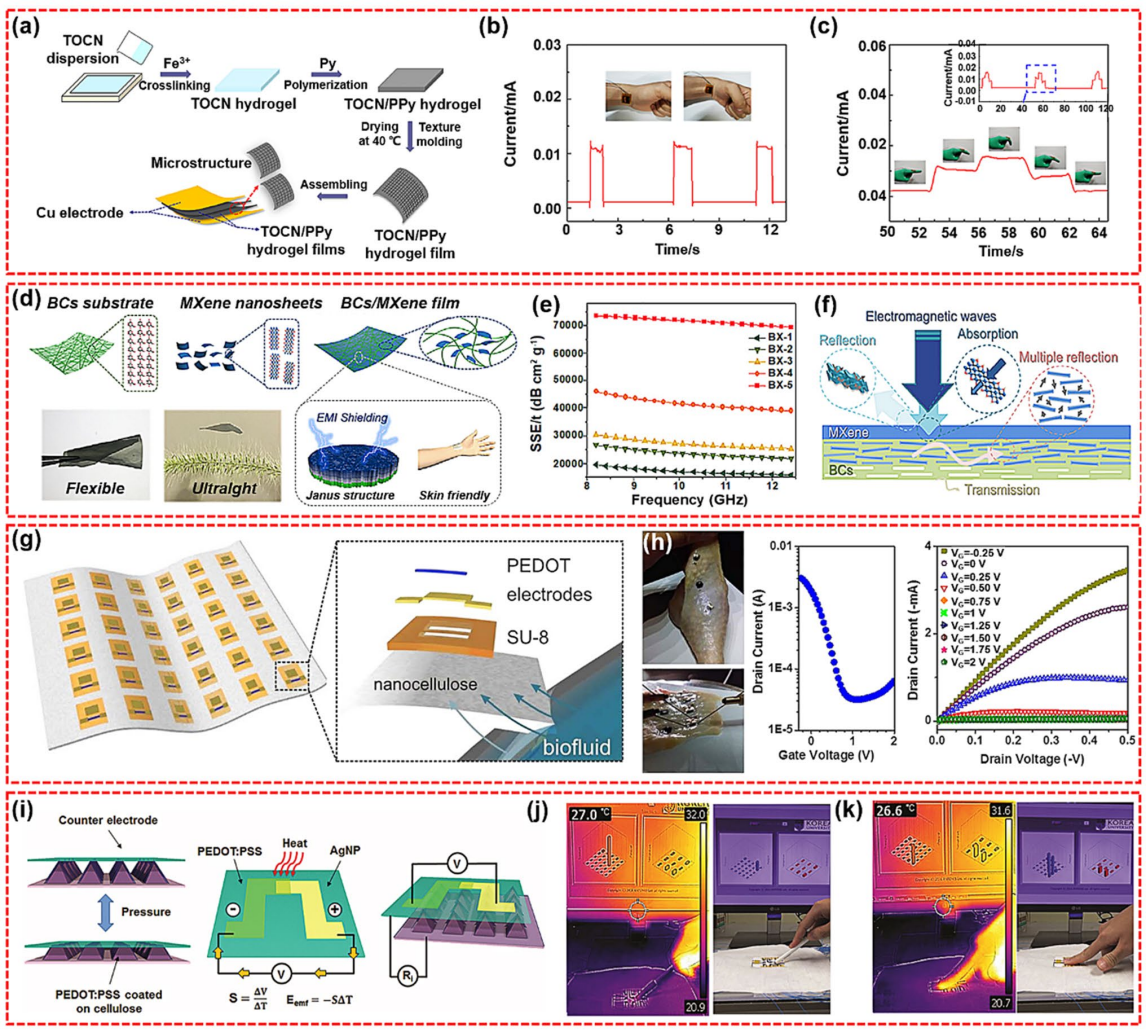
**a** Schematic illustration of the flexible E-skin preparation. **b** Real-time I-t curves of wrist bending, and **c** finger bending [124]. **d** Schematic representation of the fabrication of the transparent bacterial celluloses/MXene film with Janus structure. **e** SSE/t value of the BCs/MXene films in the X-band region. **f** Schematic illustration of the EMI wave shielding mechanism of BCs/MXene film [125]. **g** Illustration of bioelectronic decal composed of a sheet of nanocellulose containing multiple OECTs, and expanded schematic of a single OECT decal. **h** OECT decal laminated to biological tissue, i.e., chicken skin, and resultant transistor performance [126]. **i** The operating principles of the nanocellulose tactile sensor to detect various physical stimuli such as temperature and pressure. Thermographic images and real-time mapping images of a bimodal sensor array; **j** when touching the sensor array by hand, it responds to pressure and temperature. **k** On the other hand, when touching it with a pen, it responds only to the pressure, and the response to temperature is insignificant [127]

Skin electronics are flexible, stretchable, and self-healing devices that can attach seamlessly to the human skin, therefore have a bright application prospect in fields of medical diagnosis, artificial intelligence, and biological studies [128–131]. Flexible CNP with the ability to adhere to various surface has been recognized as promising candidates for preparing electronic skins. Gao et al. introduced PPy into TEMPO-oxidized CNFs (TOCN) through in situ polymerization and then prepared TOCN/PPy electronic skin with surface microstructure with nylon gauze as microstructure template, as shown in Fig. 4a [124]. The prepared electronic skin exhibited excellent sensing and mechanical properties. The signal is detected in Fig. 4b when the volunteer performed a wrist bending exercise. In addition, when the wrist was bent at a certain angle, the output signal stabilized at a constant value. As shown in Fig. 4c, the electronic skin was fixed at the finger joints, and when the fingers were bent at different angles, different output signals were detected. Therefore, TOCN/PPy electronic skin had broad application prospects in health monitoring and human–computer interaction. Gao et al. developed an all paper-based piezoresistive (APBP) pressure sensor through a facile, cost effective, and environmentally friendly method [132]. This pressure sensor was based on a tissue paper coated with AgNWs as a sensing material, a nanocellulose paper (NCP) as a bottom substrate for printing electrodes, and NCP as a top encapsulating layer. Furthermore, the APBP sensor had been mounted on the human skin to monitor physiological signals (such as arterial heart pulse and pronunciation from throat) and successfully applied as a soft electronic skin to respond to the external pressure.

MXene is a type of two-dimensional transition metal carbides and/or nitrides, with a large aspect ratio and metal conductivity, and is widely studied and considered as a potential wearable material. Yang et al. prepared Ti_3_C_2_T_x_/BC film to construct a pressure sensor [133]. The prepared sensor device exhibited high mechanical strength (225 MPa), high sensitivity (up to 95.2 kPa^−1^, in < 50 Pa region), and fast response (95 ms). For the practical application demonstration, the sensor can collect tactile signals of different gestures, including holding the cylinder, expanding five fingers, pinching, and holding the mouse. In addition, the sensor can also monitor a variety of other human biological activities (such as swallowing, heartbeat, pulse, acoustic vibration, and gesture motion) and can be used as an electronic skin to map the pressure distribution, clarifying its potential applications in medical diagnosis, intelligent robot, and human–computer interaction. Ma et al. developed a BC/MXene film with Janus structure as a skin contact material (Fig. 4d) [125]. BC act as a substrate and frame to effectively capture the infiltrated MXene nanosheets, rather than simply mixing, leading to the formation of the Janus structure. The MXene-based composite film also achieved a super high SSE/t of ∼ 69,455.2 dB cm^2^ g^−1^ at a thickness of approximately 1.732 μm (Fig. 4e). The potential EMI attenuation mechanisms of BCs/MXene films are presented in Fig. 4f. It may hold the promise for broad applications of such BCs/MXene films in wearable smart electronic devices.

Additionally, Yuen et al. reported a self-adhesive ultra-thin electronic biological sticker that can collect, transmit, and detect biological fluids [126]. The device consists of a thin film organic electrochemical transistor (OECT) fabricated on a thin (< 20 μm) porous microbial nanocellulose membrane (Fig. 4f). As shown in Fig. 4g, the OECT sticker was tested on chicken skin moistened with simulated sweat to evaluate the robustness of the device performance on biological tissues with rough local contours. The results showed that the transistor performance was comparable to other devices tested, and the mA-level current and ION/IOFF ratio were close to three orders of magnitude. The uniform performance of OECT stickers on various surfaces indicated the robustness, flexibility, and stability of OECT stickers, which indicated a good prospect for future biomedical applications. Interestingly, Jung et al. reported a fully integrated vertically stacked nanocellulose tactile sensor capable of sensing temperature and pressure at the same time [127]. Figure 4h shows the relative resistance change in the pressure sensor, which had a micro-pyramid structure, principles of the thermoelectric-based temperature sensor, and the structure of tactile sensor. The authors evaluated the operation of the sensor using real-time mapping arrays and thermal images, as shown in Fig. 4i-j. When the sensor array was touched by hand, it responded to pressure and temperature. On the other hand, when touched with a pen, it only reacted to pressure, but not to temperature. The results showed that the nanocellulose-based sensor array has high sensitivity, fast response, negligible interference, and durable performance and had important research value in the fields of artificial intelligence equipment, electronic skin, and robots.

#### Organic Light-emitting Diodes

**Fig. 5:**
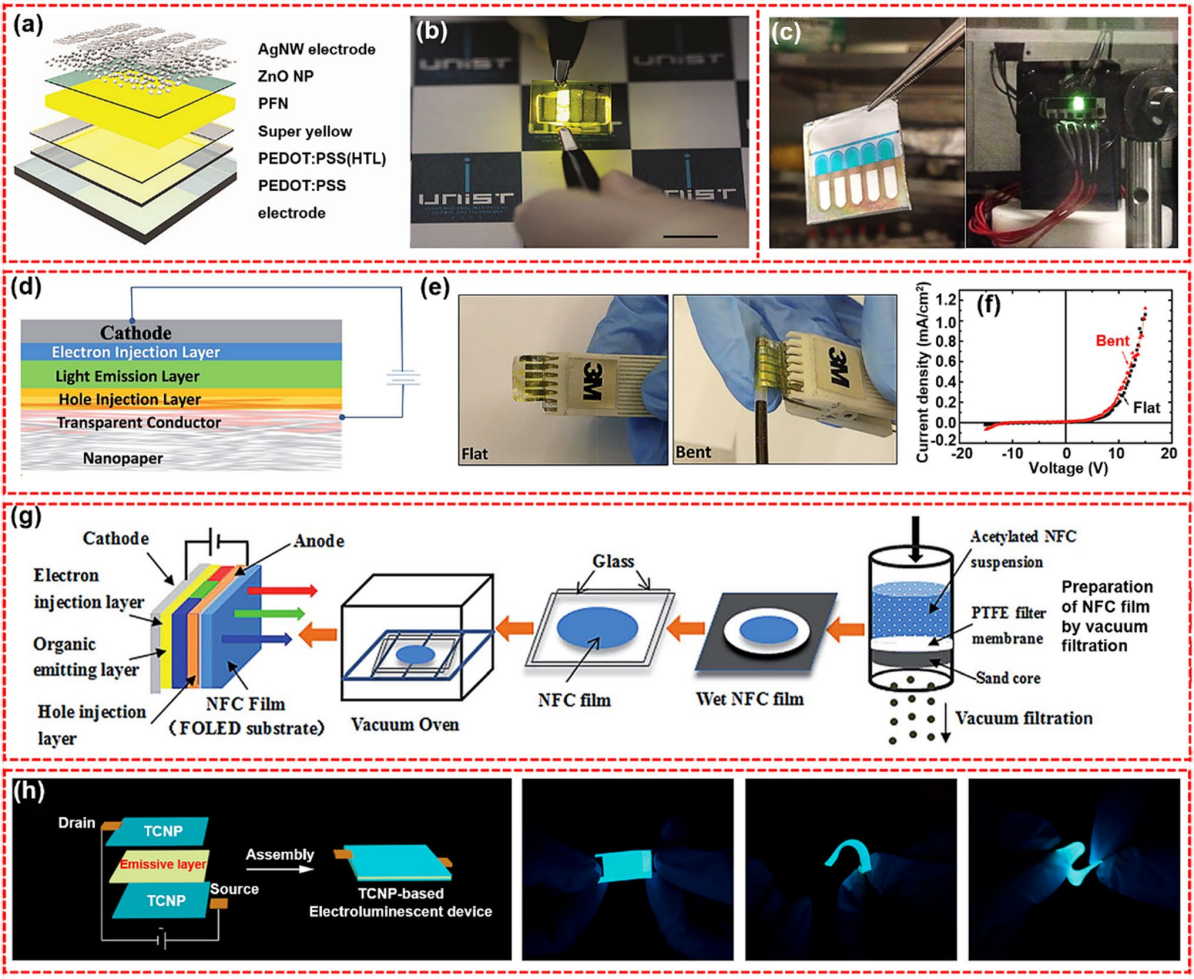
**a** Schematic structure of the TOLED on the CNCs-based hybrid film and **b** photograph of the TOLED on the hybrid substrate emitting light through bottom direction [134]. **c** Photograph of five inverted top-emitting OLEDs on a CNCs substrate mounted on glass (left), and an illuminated OLED on CNCs substrate (right) [135]. **d** Schematic drawing of a nanopaper OLED device. **e** Pictures of the flat and bent nanopaper OLED device. **f** *J-V* curve of the flexible OLED in the flat and bent states, respectively. The bending radius is 1.5 mm [136]. **g** Preparation of NFC film as a FOLED substrate [137]. **h** Schematic illustration of TCNP-based flexible electroluminescent devices; photographs of the flexible TCNP-based electroluminescent devices with horizontal, curved, and “Z-shaped” folding, which exhibited high luminous brightness and flexibility [138]

Organic light-emitting diodes (OLED) are light-emitting devices in the form of all-solid film. OLEDs have advantages of lightweight, low power consumption, long life, and good flexibility. Figure 5a-b illustrates a schematic diagram of the device structure. Flexible substrate is a very important part of electronic displays, and its constituent materials are required to have excellent mechanical properties, thermal stability, flexibility, and smooth surface. As shown in Fig. 5c, Najafabadi et al. sequentially deposited the cathode layer (Al and LiF), organic layer (TpPyPB), emissive layer (CBP:Ir(ppy)_3_), and anode layer (MoO_3_ and Au) on a CNCs-based CNP substrate to assembly a OLED with a maximum brightness of 74,591 cd m^−2^ [135]. The obtained OLED is soluble in water, which helps solve the problem of electronics recycling difficulties. Nanopaper has the potential to play an important role in various emerging electronic devices. However, compared with plastic or glass counterparts, nanopaper has some drawbacks that limit its practical application in the electronic field: (1) the relatively high surface roughness, (2) high water absorption, (3) low decomposition temperature, and (4) high material cost. Zhu et al. compared several properties of nanopaper, regenerated cellulose film, and traditionally used flexible plastics as flexible electronic substrates (Fig. 5d) [136]. Nanopaper had a much higher haze value and was an ideal choice for low-glare displays and solar cells. As shown in Fig. 5e–f, OLED devices prepared on nanopaper have stable performance in both flat and bend states. Yang et al. prepared a CNP from the acetylated CNFs and used it as OLED substrates, as shown in Fig. 5g. The effects of the degree of acetylation on the film properties related to the OLED performance, specifically the film homogeneity, flexibility, thermal stability, transmittance, and mechanical properties, were carefully investigated [137]. Zhang et al. prepared a highly transparent and conductive nanopaper (TCNP) by conducting in situ photopolymerization of conductive polymerizable deep eutectic solvent (PDES) monomer on a nanopaper substrate made of CNFs (Fig. 5h) [138]. TCNP also showed excellent optical and electrical durability after more than 6000 bend recovery at a bending angle of 150°.

### Energy Conversion and Storage Devices

#### Supercapacitors

**Fig. 6:**
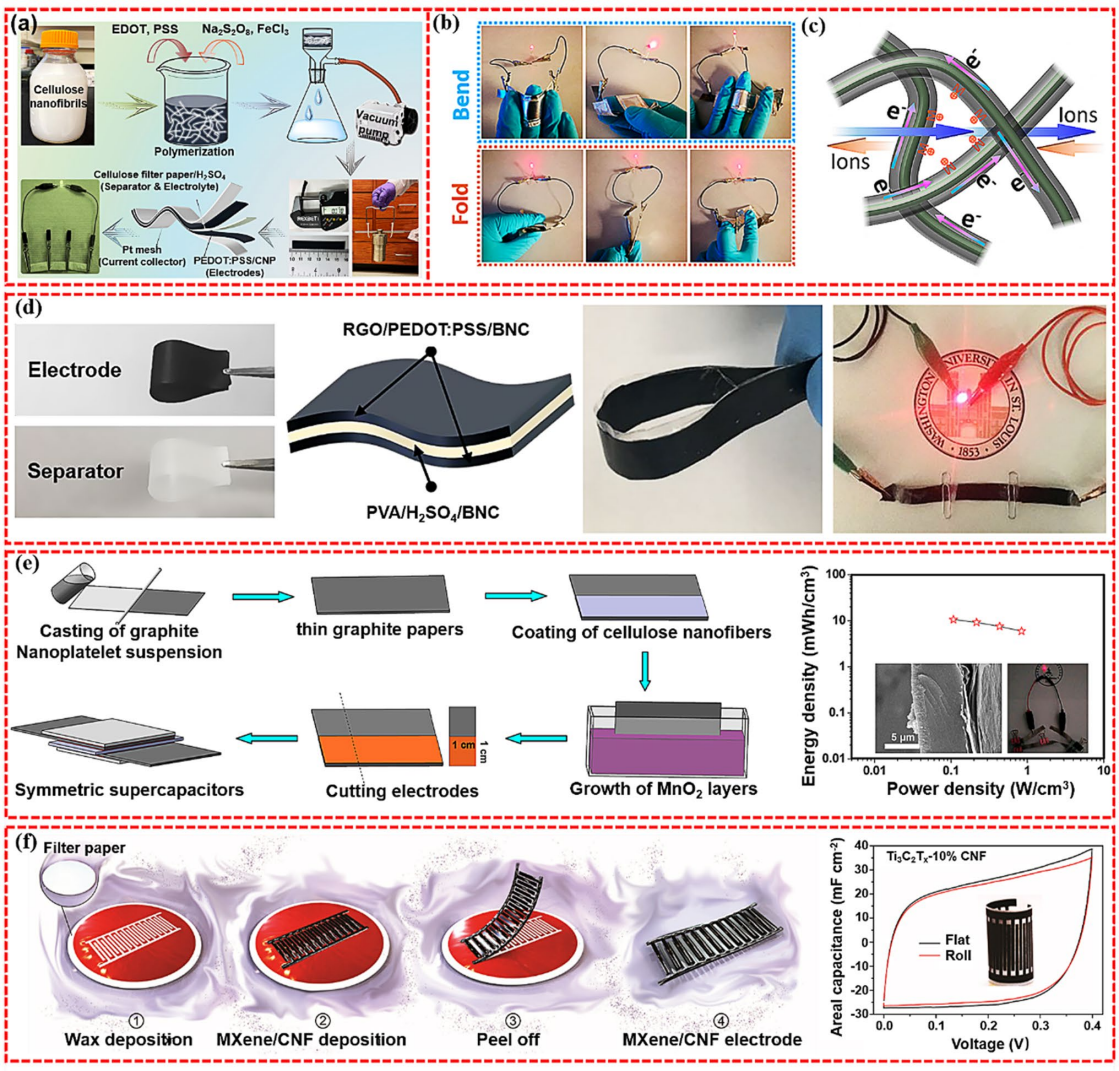
**a** Schematic illustration of the preparation of PEDOT:PSS/CNFs and the fabrication of PEDOT:PSS/CNP [144]. **b** Photograph of LED powered by the devices in series under different deformations. **c** Schematic diagram showing the charge transfer and electrolyte ion transport in the nanofibrous and conductive networks of CNF@c-MOF nanopapers [146]. **d** Photographs showing the flexibility of the components (electrode and separator) of device and a schematic showing the structure of the solid-state supercapacitor device based on RGO/PEDOT: PSS/BNC electrodes. Optical image showing the flexibility of assembled supercapacitor. LED indicator lighted by assembled device [145]. **e** Schematic diagram of the fabrication procedures of the electrodes from thin graphite papers grown with MnO_2_ layers and the electrochemical performance of the symmetric supercapacitor [147]. **f** Schematic illustration showing different fabrication steps for Ti_3_C_2_T_x_/CNFs-based micro-supercapacitors and CV curves at a flat and roll status of Ti_3_C_2_T_x_-10% CNFs-based micro-supercapacitors [150]

Supercapacitor is a type of energy storage device, which is generally composed of cathode, anode, electrolyte, and separator. Comparing with batteries, supercapacitors have the advantages of high-power density, fast charging/discharging speed, and long cycle life [139, 140]. Nanopaper has excellent mechanical properties and good flexibility, therefore an excellent matrix material for the manufacturing of flexible electrodes. Cellulose is inherently non-conductivity. Integrating CNP with conductive materials possessing high electrical conductivity would efficiently resolve this issue. Li et al. prepared flexible and lightweight paper electrodes using polyaniline (PANI)-modified BC through a simple vacuum filtration method [141]. The results showed that the electrode had excellent specific capacitance (the specific capacitance was 656 F g^−1^ at 1 A g^−1^) and cycling stability (the specific capacitance remained 99.5% after 1000 charges). Wang et al. designed a flexible electrode with excellent mechanical properties by coupling nanocellulose with flexible PPy/graphene oxide (GO) composites [142]. The obtained electrode realized the rapid charge and discharge with a volumetric capacitance up to 198 F cm^−3^ and demonstrated good cycling stability. Zheng et al. designed a similar electrode by several steps involving vacuum filtration of CNFs and graphene nanosheets to prepare substrates, in situ polymerization of aniline to create composite electrodes, and finally assembly of all-solid-state supercapacitors [143]. Recently, several attempts have been made to fabricate PEDOT-based flexible electrodes by using CNFs as building blocks. For example, Du et al. reported a conductive PEDOT:PSS/CNFs paper electrodes for flexible supercapacitors with superior areal capacitance and cycling stability (Fig. 6a) [144]. The supercapacitor could deliver the maximum areal specific capacitance of 854.4 mF cm^−2^ (corresponding to 122.1 F cm^−3^) at 5 mV s^−1^ and offer the highest areal energy density of 30.86 μWh cm^−2^ (corresponding to 4.41 mWh cm^−3^). Jiang et al. developed a new and simple method for the preparation of RGO/PEDOT:PSS/BNC flexible electrode by adding GO thin slices and conducting polymer PEDOT:PSS in BNC matrix and then reducing GO by water treatment in the process of BNC mediated hydrogel growth, as shown in Fig. 6e [145]. The prepared electrode had excellent electrochemical performance (373 F g^−1^ at 1 A g^−1^) and cycle stability (approximately 85% capacitance retention rate in 1000 cycles). In addition, the lightweight device showed remarkable mechanical flexibility, performance stability under extreme mechanical deformation, and a capacitance retention rate of approximately 88% over 4500 cycles. Zhou and co-workers reported a highly flexible, hierarchical porous, conductive nanopapers electrode of CNFs@conductive MOF (CNF@c-MOF) for high performance supercapacitor [146]. The nanopaper electrodes and obtained supercapacitor both showed good electrochemical performances due to the smooth electrolyte transport and charge transfer of CNF@c-MOF film (Fig. 6b-d). As shown in Fig. 6f, Qi et al. easily achieved the thickening of the MnO_2_ layer by coating cellulose nanofibers on thin graphite paper [147]. The symmetrical supercapacitor assembled from optimized paper electrodes showed an extremely high volumetric energy density of 10.6 mWh‧cm^−3^ at a power density of 0.11 W cm^−3^. Another notable benefit of using CNP as the substrate was its ability to facilitate the dispersion of electroactive materials (e.g., graphene, GO, and nanotubes). This was achieved through the interaction of hydrophobic region of conductive NP (e.g., CNT and graphene) and specific crystalline faces of cellulose [148, 149], forming the hybrid complex to prevent the agglomeration or re-stacking of the electroactive particles, and in turn, promoting electrical conductivity and cycling stability of the supercapacitors. For example, Tian et al. reported that MXene (Ti_3_C_2_T_x_) colloid was stably dispersed by CNFs, and nanocomposite film electrodes with independent and good mechanical properties were assembled by using stable dispersion [150]. In addition, due to the enhanced mechanical properties of Ti_3_C_2_T_x_/CNFs electrode, the prepared micro-supercapacitor device can be bent and crimped without significantly reducing its charge storage performance, as shown in Fig. 6g.

#### Fuel Cells

**Fig. 7:**
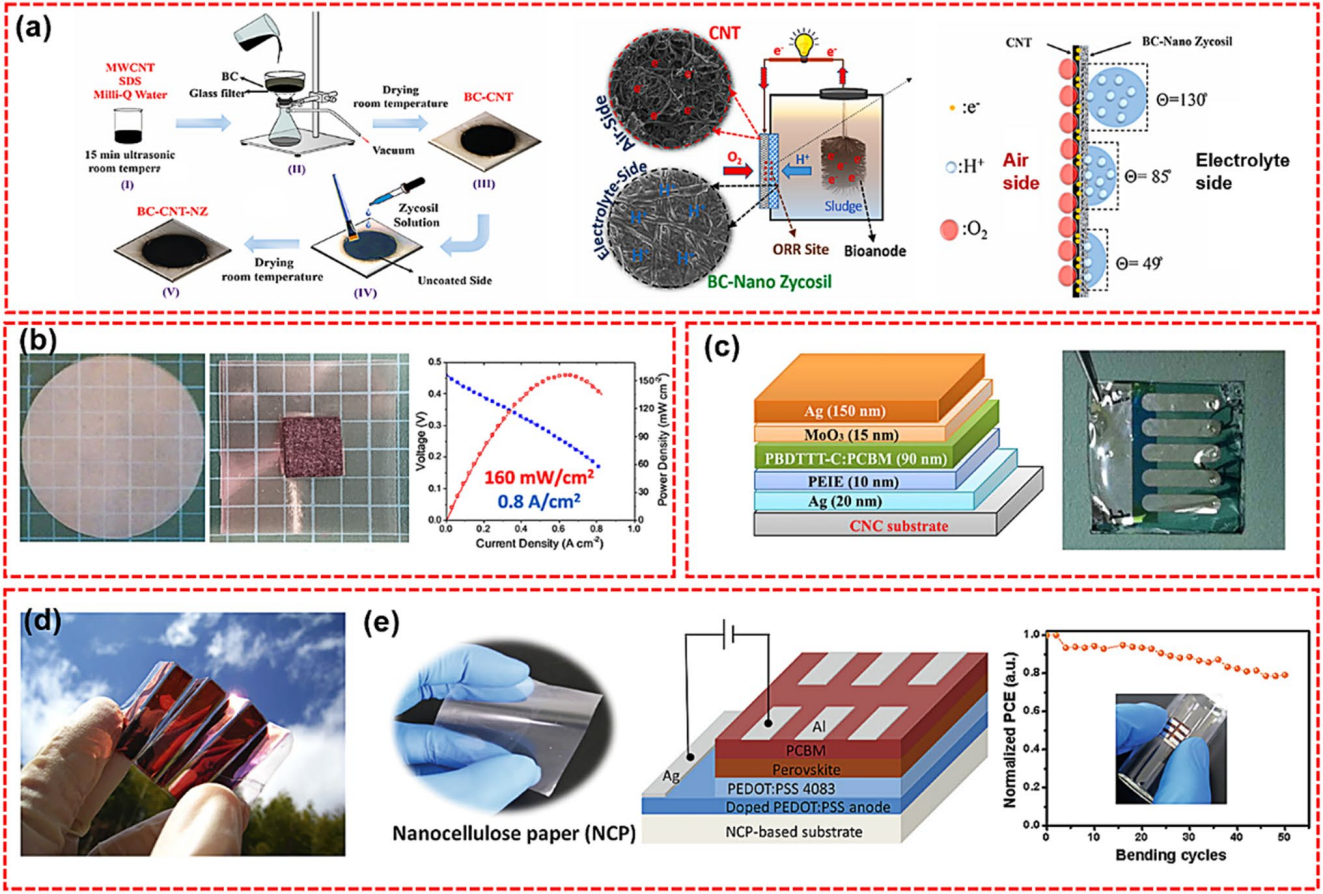
**a** The preparation process of BC-CNT-NZ, the detailed configuration of SCMFC and its compartments, and the role of BC-CNT-NZ in SCMFC [152]. **b** Photographs of S-CNFs paper, paper fuel cells and the power density of paper fuel cells [153]. **c** Schematic structure of the organic solar cell on CNCs substrates (left) and image of an assembled solar cell (right) [111]. **d** Portable paper solar cells based on foldable and lightweight transparent conductive nanofiber paper [106]. **e** NCP and the device performance of NCP-based PSCs [156]

Fuel cells are the most promising source of green energy for road transportation, which convert chemical energy into electricity with an efficiency of up to 80%. The proton membrane is an essential part of the fuel cell and must have high proton conductivity to achieve high performance. Nafion^®^ made of poly (perfluorinated sulfonic acid) is one of the most widely used polymer membranes for fuel cells owing to its superior thermal stability, mechanical properties, and proton conductivity. However, there are some disadvantages associated with the application of Nafion such as high production costs, complex synthesis processes, toxic intermediates, and reduced proton conductivity at high temperature. CNP, as a new type of biodegradable and inexpensive ionomer film, is suitable to manufacture proton membrane for polymer-electrolyte fuel cells and methanol fuel cells. An et al. used a simple solution method to mix the CNCs/imidazole (Im) hybrid into the Nafion matrix to prepare the Nafion/CNCs/imidazole (NCI) composite material, and then studied the effect of CNCs/Im on the performance of the NCI membrane [151]. The results demonstrated that proton conductivity of the NCI membranes changes according to the content of Nafion 117, and reached the highest value of 6.19 × 10^–4^ S m^−1^ with an Nafion 117/(CNCs/Im) ratio of 2:1. Mashkour et al. fabricated a new monolithic membrane electrode assembly (MEA) composed of BC, CNT, and nano-zycosil (NZ) and used it as the air cathode in a single-chamber MFC (SCMFC) (Fig. 7a) [152]. As a kind of nanocellulose with oxygen barrier properties, BC can maintain the anaerobic conditions of the anode compartment. For the MEA, protons crossed BC- NZ to meet electrons and oxygen at the BC-CNT interface. High water contact angle (WCA) (130°) decreased the total protons reaching the BC-CNT interface and therefore made a limiting factor to oxygen reduction reaction (ORR). While the low WCA (49°) resulted in leakage and, therefore, lower concentration of oxygen in ORR sites (because of lower soluble oxygen in the water phase rather than air). Thus, a balanced WCA (85°) not only provided a sufficient proton transfer in the reaction sites by BC but also prevents leakage by NZ coating. A balanced WCA presented better electrochemical performance for the MEA. Compared with commercial gas diffusion electrode (GDE), the resistance of MEA was significantly reduced. This finding was attributed to the uniform connection between BC, CNT, and NZ and the high conductivity of CNT. In addition, the capacitance of MEA (65 mF) was much higher than that of GDE (0.73 mF). Therefore, compared with GDE, MEA showed higher performance and lower cost in SCMFC power generation and wastewater treatment. Bayer et al. synthesized a sulfonated cellulose nanofiber (S-CNFs) membrane and successfully introduced it into a "paper fuel cell" as a substitute for Nafion (Fig. 7b) [153]. The resulting paper fuel cell had high current density (> 0.8 A cm^−2^) and power density (156 mW cm^−2^) under standard measurement conditions (H_2_/air; 80 °C; 95% RH; 0.1 MPa), which was due to the enhanced conductivity of S-CNFs film. Although it is difficult to directly compete with mature (but more expensive) ionomers such as Nafion, S-CNFs polymer electrolyte fuel cells (PEFC) are cheaper and open a new way for the commercialization of PEFC without Nafion. Jiang et al. used two different acid modifications to improve proton conductivity to prepare a new type of proton conductive polymer electrolyte membrane which was used in fuel cells [154]. One was a conventional inorganic acid, phosphoric acid (H_3_PO_4_), and the other was an unusual organic acid, phytic acid (PA). In particular, the membrane electrode assembly fabricated with H_3_PO_4_/BC and PA/BC membranes reached the initial power densities of 17.9 and 23.0 mW cm^−2^, which were much higher than those reported in the literature in a real H_2_/O_2_ fuel cell at 25 °C.

#### Solar Cells

Solar cells can convert the inexhaustible solar energy into electricity or heat [123]. However, due to the high cost of collector system and the low efficiency of energy conversion, the solar energy harvesting is still restricted in limited circumstances [123]. Adopting low-cost solar cell substrates is a feasible way to overcome this challenge. Nanopaper is considered as a promising green substrate for solar cell construction due to its excellent mechanical properties, adjustable optical properties, and low cost [43]. Hu et al. reported a transparent nanopaper-based solar cells, but the photoelectric conversion efficiency was only 0.21% [155]. To improve the conversion efficiency, Zhou et al. employed CNCs-based CNP possessing smoother surface (Fig. 7c) [111], observing a notable improvement in conversion efficiency of 2.7%. More importantly, the results shown that these solar cells can be easily separated into their major components using low-energy processes at room temperature, opening the door for a truly fully recyclable solar cell technology which demonstrated great recyclability. As shown in Fig. 7d, Nogi et al. reported an optically transparent conductive paper made of CNFs and AgNWs [106]. The results shown that the optical transparency and conductivity of the conductive paper were comparable to that of indium tin oxide (ITO) glass. This paper-based solar cell exhibited a high-power conversion efficiency of 3.2%, which is equivalent to an ITO glass-based solar cell. Due to the high affinity and high degree of entanglement between CNFs and AgNWs, the CNP maintained high conductivity, and the paper solar cell can still generate electricity during and after folding. Gao et al. developed transparent nanocellulose paper (NCP) with coating of acrylic resin as substrates to fabricate flexible perovskite solar cells (PSCs), which were biodegradable and easily disposable (Fig. 7e) [156]. The power conversion efficiency (PCE) of PSC based on NCP reached 4.25%. In addition, the flexible PSC exhibited good stability and maintains > 80% of the original efficiency after 50 bends. This low-cost, biodegradable NCP-based substrate with acrylic resin coating was also suitable for other functional devices, which can promote the development of flexible electronics. Additionally, Wu et al. proved that CNP can improve the efficiency of organic solar cells (OSC) and effective wide-angle light capture [19]. The highest power-conversion efficiency of the fabricated OSC reached up to 16.17%, which was much higher than that without CNP as the substrate. Although substantial efforts have been devoted to fabricating nanopaper-based solar cells, there are still many obstacles to the practical application of CNP-based solar cellulose, among which, the short service life of nanopaper-based solar cells is one of the most imperative.

#### Nanogenerators

**Fig. 8:**
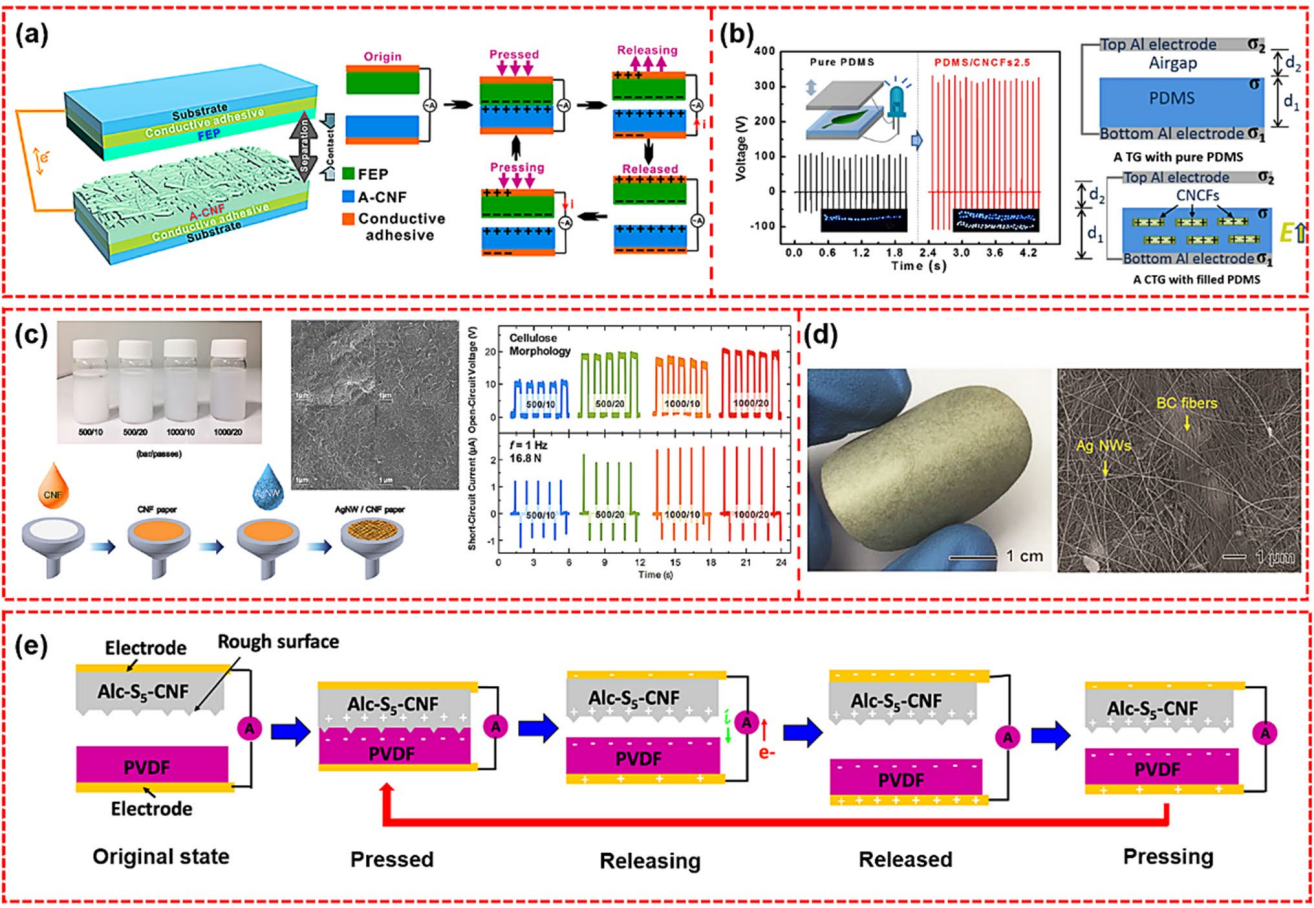
**a** Structure of a TENG that uses the A-CNFs film as a contact material, working principle of the A-CNFs-based TENG, and stability and durability test of the TENG after 10,000 cycles [161]. **b** Performance comparison of pure PDMS and PDMS/CNCFs, and illustration of the triboelectric contact electrification processes with the net electric field E in composite films along the direction from the PDMS to the top Al electrode [162]. **c** CNFs paper: CNFs dispersions homogenized at 500 bar and 1000 bar and different numbers of passes (10, 20). Schematic of AgNWs/CNFs paper fabricated using a vacuum-filtration technique. SEM images of cellulose surface with different morphologies. And electrical output of TENG (open-circuit voltage and short-circuit current) depending on cellulose morphology [163]. **d** Photograph and SEM image of the BC composite paper, and picture showing an electric circuit and an array of 200 lit LED bulbs during the circuit operation by the large-area BC-TENG [165]. **e** Schematic illustration of the operating principle of the TENG device [166]

Nanogenerators convert mechanical energy into electrical energy, making them ideal to fuel portable electronic devices as an alternative power source [157]. According to the power generation principle, nanogenerator is generally divided into two types: triboelectric nanogenerator (TENG) and piezoelectric nanogenerator (PENG) [158]. Yao et al. developed a flexible and transparent TENG material by pairing nanopaper as the friction layer with fluorinated ethylene propylene [159]. The high surface roughness of nanopaper provides a large surface area for contact and electrostatic charge generation. Recently, Kim et al. developed a BC-based TENG composed of Cu/BC composite nanopaper and Cu foil, yielding cumulative charge and peak power density of 8.1 μC m^−2^ and 4.8 mW m^−2^, respectively [160]. Nie et al. performed simple aminosilane modification on CNFs and prepared an A-CNFs-based flexible TENG with increased frictional charge density. Figure 8a shows the structure of TENG using A-CNFs film as the contact material and the working principle of TENG based on A-CNFs [161]. It was found that the voltage of TENG based on A-CNFs can be maintained at an almost constant level within 10,000 cycles, which indicated that its output performance was relatively stable and can provide sufficient voltage for electronic devices. Peng et al. developed a new type of bio-renewable cellulose composite friction generator (CTG) with stable power output performance [162]. The new CTG using polydimethylsiloxane (PDMS) matrix and oriented cellulose nanochips (CNCFs) as effective dielectrics showed enhanced triboelectric performance, generating power 10 times that of pure PDMS film-based TENG, and can instantly light up as many as 100 multi-color commercial LEDs (Fig. 8b). Kim et al. developed a flexible all-cellulose-based TENG. As shown in Fig. 8c, a double-layer film suitable for all cellulose-based TEN can be prepared in only 20 min via vacuum filtration [163]. It was found that the cellulose morphology can affect the electrical output of TENG (open-circuit voltage and short-circuit current). The higher pressure and more passes of filtration in fabricating CNFs, the electrical outputs of the fabricated TENG were enhanced due to the increased effective contact area between CNFs side of upper paper and AgNW side of bottom paper. The results showed that the electrical outputs were maximized in case of 0.1 wt% of the AgNWs and 1000 bar/20 passes CNFs paper, corresponding to a peak open-circuit voltage of 21 V, a short-circuit current of 2.5 μA, and a power density of 693 mW m^−2^ for 10 MΩ of external resistance. Zhao et al. developed a piezoelectric composite nanopaper composed of BaTO_3_ NP and BC, and yielded good electrical properties [164]: the output voltage was 14 V; the peak current density was 190 nA cm^−2^; and the maximum power density was 0.64 μW cm^−2^. In addition, as shown in Fig. 8d, Oh et al. prepared a high-conductivity ferroelectric BC composite paper containing AgNWs and BTO NP through simple vacuum filtration, and successfully used it for the development of large-area, high-performance TENG [165]. Interestingly, Roy et al. revealed for the first time the powerful power of allicin extracted from garlic juice in enhancing the triboelectric properties of CNFs to produce high-performance TENG. Figure 8e showed the working principle of the TENG device [166]. It has been found that after allicin grafting, the mechanical strength of the CNFs membrane was stronger due to the higher degree of intermolecular and intramolecular fiber–fiber hydrogen bonds. In addition, allicin grafting rich in high dipole sulfoxide groups (–S=O) results in the CNFs membrane with high friction positiveness, so when coupled with the friction negative material PVDF, high performance TENG was realized. As a result, the peak output voltage and current reached 7.9 V and 5.13 µA, respectively, which was 6.5 times higher than that of pure cellulose-based TENG (0.80 µA, 1.23 V). Compared with pure cellulose-based TENG, the power density was also increased by 41 times. This work provided new insights for the design of next-generation sustainable energy harvesting devices. However, the output of the nanopaper-based PENG is much lower than that of the nanopaper-based TENG. PENG uses the piezoelectric effect to effectively collect micromechanical energy and convert it into electrical energy to achieve energy harvesting. For example, Wu et al. prepared TOCN/MoS_2_ composite membrane and applied it in PENG [167]. The one-dimensional CNFs were intercalated between the layers of MoS_2_ nanosheets to form a “brick–mortar structure,” resulting in the improved properties of the nanocellulose films. The obtained TOCN/MoS_2_ PENG output a maximum open-circuit voltage of 4.1 V and a short-circuit current of 0.21 μA, which were nearly 3 times higher than those of the neat TOCN PENG.

### Packaging Materials

Considerable efforts have been devoted to developing bio-based packing materials to address environmental problems associated with petroleum-based packaging materials [168]. CNP has excellent barrier performance to minimize the penetration of oxygen, other gases, and volatile compounds, thus is a suitable packing material to increase the shelf-life of foods and prevent unnecessary odor accumulation or contamination [169]. Additionally, some desirable traits for packing materials could be achieved by functionalizing CNP with other materials [170]. For example, Vähä-Nissi et al. used TEMPO-oxidized CNFs coating and bio-based polyethylene to prepare the barrier layer of a multilayer packaging film [171]. The bio-based multilayer film provided by CNFs had an oxygen barrier and was suitable for demanding food and modified atmosphere packaging (MAP). During the storage time used in this study, the MAP bags made of these multilayer films maintained their atmosphere and shape and prevented the crushed hazelnuts from further oxidation. Leite et al. prepared transparent gelatin/rosin-grafted CNCs (Gel/r-CNCs) by solution casting composite film for antibacterial packaging [172]. Gel/r-CNCs film had moderate water vapor permeability (0.09 g mm/m^2^ h kPa), high tensile strength (40 MPa), and Young's modulus (1.9 GPa). Compared with traditional CNCs, the addition of r-CNCs can improve the optical properties, water vapor barrier properties and tensile properties of the gelatin film. Currently, the major obstacle of CNP for packaging application is its poor water resistance. In order to address this problem, various hydrophobic modification strategies have been proposed, such as esterification, polymer grafting, plasma fluorination, among others. For example, Oberlintner and co-workers reported an ultrafast approach to improve the CNP surface hydrophobicity by cold plasma fluorination [173]. Specifically, the CNP sample was exposed to CF_4_ plasma for less than 10 s, and the contact angle of the treated CNP significantly increased from 46° to 130° after only 30 s of plasma processing.

### Water Treatment

**Fig. 9:**
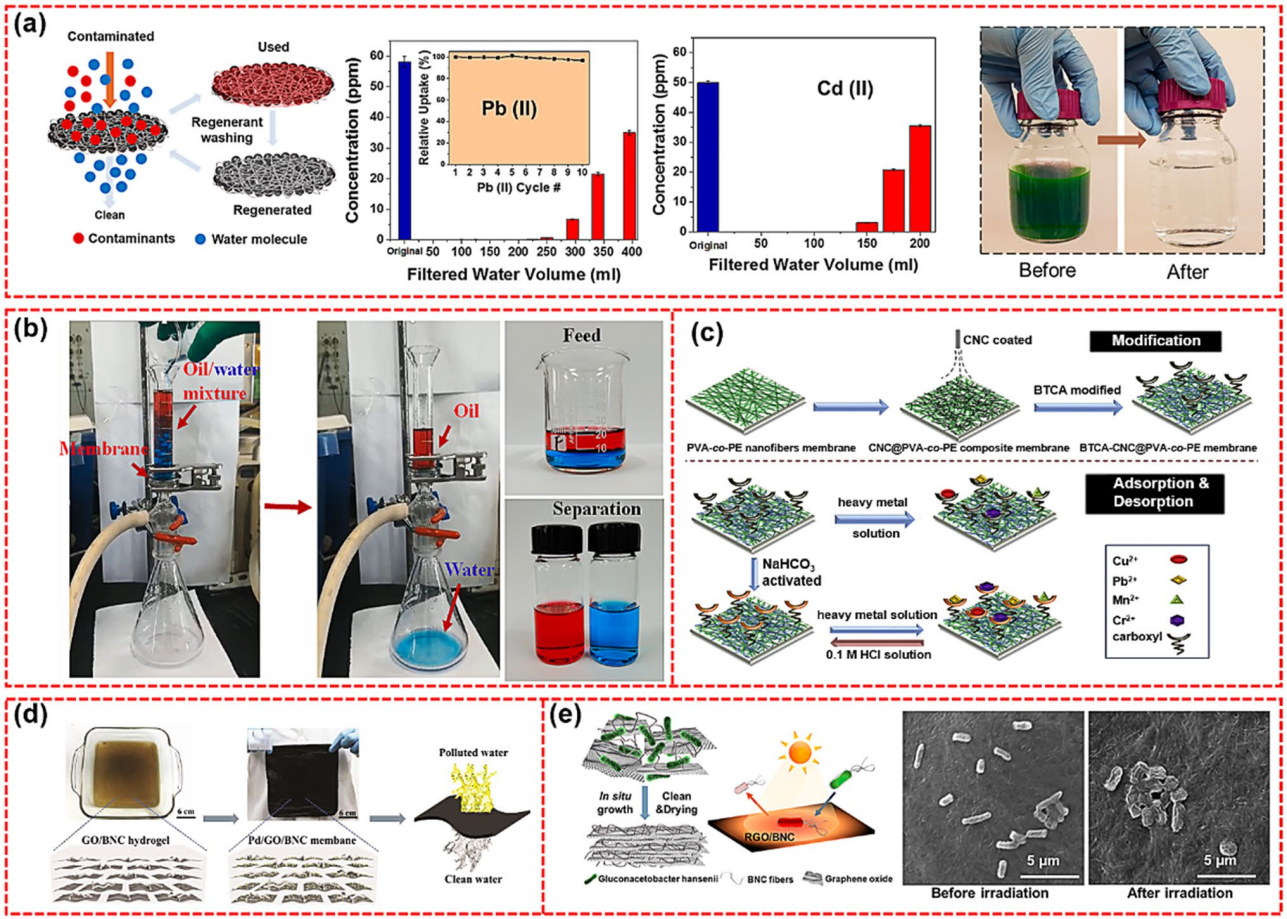
**a** Schematic illustration of membrane filtration experiment [179]. **b** The O/W separation tests: a mixture of oil and water was added into the filtration device equipped with the BSn@P membrane, the water was permeated while oil could not pass through the membrane [181]. **c** Process of BTCA modified nanofibrous membrane as heavy metal removal [180]. **d** Schematic illustration and photographs showing the procedures of Pd/GO/BNC membrane for water purification [177]. **e** Schematic diagram of photothermally activity reduced GO/bacterial nanocellulose biofouling-resistant fouling ultrafiltration membrane, and SEM images of E. coli on RGO/BNC membranes before and after irradiation [182]

Developing simple and efficient methods to purifying contaminated water is of high significance and demand. Various technologies have been developed to purify polluted water, including reverse osmosis membrane, solar evaporation, membrane filtration, etc. [174, 175]. Recently, membrane filtration technology has attracted increasing attention because of its high efficiency, good chemical stability, and small impact on the environment. It has been reported that various contaminants such as heavy metal ions, viruses, organic solvents, pesticides, and herbicides could be effectively removed by the simple membrane filtration method [176]. CNP can be used for water treatment because of its rich chemical functionality, small pore size, and excellent mechanical properties (Fig. 9d) [177]. Ma et al. developed a novel ultrafiltration membrane with a maximum pore size of 55 nm using CNFs nanopaper as the top barrier layer, PAN electrospun scaffold as the intermediate layer, and PET nonwoven fabric as the supporting substrate [178]. Hamed et al. prepared a new type of membrane composed of polydopamine (PDA) particles and BNC, which can effectively remove various metal ions and organic dyes in polluted water (Fig. 9a) [179]. Zhu et al. designed a 1, 2, 3, 4-butanetetracarboxylic acid (BTCA) modified composite membrane for heavy metal ion adsorption [180]. As shown in Fig. 9c, the composite membrane was composed of CNCs and PVA-co-ethylene. To improve the adsorption rate, NaHCO_3_ was used to activate the BTCA modified membrane. The results showed that the modified nanofiber membrane showed good adsorption performance for single metal ion and metal ion mixture after activation by NaHCO_3_, and the equilibrium adsorption capacity could reach 471.55 mg g^−1^ at 15 ℃. In addition, after three times of repeated use, the composite membrane was found to be stable, indicating that the NaHCO_3_ activated membrane was a good material for removing heavy metal ions. Wahid et al. designed a superhydrophilic/underwater superhydrophobic (SUS) membrane for oil/water (O/W) separation by blending BC nanofibers with silica particles (SiO_2_ MPs) followed by self-polymerization of bio-inspired PDA [181]. As shown in Fig. 9b, the composite membrane shows excellent O/W separation ability with a separation efficiency of 99.9%, showing its potential for oily wastewater treatment in the environmental field. Jiang et al. prepared an anti-biological pollution ultrafiltration membrane based on reduced graphene oxide (RGO) and BNC [182]. As shown in Fig. 9e, the ultrafiltration membrane had excellent photothermal properties and showed effective bactericidal activity under light. Karim et al. developed CNCs and CS composite membranes via freeze-drying followed by compaction. The obtained membrane with a pore size of 13–10 nm has a low water flux of 64 L (m^2^ h)^−1^ [183].

## Summary and Outlook

In this review, we summarized several main methods to construct CNP and key parameters governing the properties of CNP. Also, we discussed various functional CNP with special properties such as magnetism, photonic property, and conductivity, and recapitulated their potential applications in various fields. Although much progress has been achieved in these areas, there are still some obstacles needed to be overcome on the way to their practical applications.

1. At present, there are three main methods including suction filtration, casting, and coating to produce CNP. Among them, the suction filtration method has been frequently used and has the most potential for scale-up production. However, it still requires complex and time-consuming processes such as dewatering, wet paper web transfer and drying, resulting in low efficiency. Therefore, much more efforts will be needed to develop manufacturing strategies for continuous, large-scale, and efficient preparation of CNP.
2. The high hydrophilicity of unmodified CNP arising from the existence of abundant hydroxyl groups greatly hinders its widespread applicability. Thus, enhancing the water resistance of CNP by either introducing suitable polymers and additives or post-treatment still need further investigation.
3. To date, a variety of approaches have been developed for functionalizing CNP by adjusting the self-assembly process or introduce extrinsic functional components. For example, the CNP can be functionalized with magnetism, antibacterial properties, electrical conductivity, and so on by introducing active materials such as magnetic nanoparticles, conducting polymers, and carbon nanomaterials. And the functionalized CNP has been applied to many emerging fields including flexible sensors, solar cells, energy storage devices, packaging materials, water treatment, among others. Nevertheless, it is still necessary to design better microscopic pore structure and surface chemistry to obtain CNP with higher performance and more functionalities.
4. Multi-disciplinary research on CNP can be carried out, such as performing foundational study on CNP microstructure through computer simulation and big data processing, developing new assembly methods and solvents, designing advanced and intelligent nanopaper structure and functionality, so as to further broaden the application of CNP. In addition to nanocellulose, it is still necessary to develop other natural polymer materials and combine the advantages of nanocellulose and other natural polymers to construct biodegradable and sustainable composites for the replacement of nonrenewable ones.
